# Supplementary material for: Comparison of COVID-19 and Non-COVID-19 Pneumonia in Down Syndrome
Source: J Clin Med. 2021 Aug 23;10(16):3748. doi: 10.3390/jcm10163748 (PMC8397064; doi:10.3390/jcm10163748)
Supplement: Supplementary file 1 [file jcm-10-03748-s001.zip › jcm-1314675-supplementary.pdf]

**SUPPLEMENTARY MATERIAL**

**Figure S1**. Age distribution of COVID-19 DS patients by survey group. (cases reported by clinicians are depicted in red and those reported by families are shown in blue)

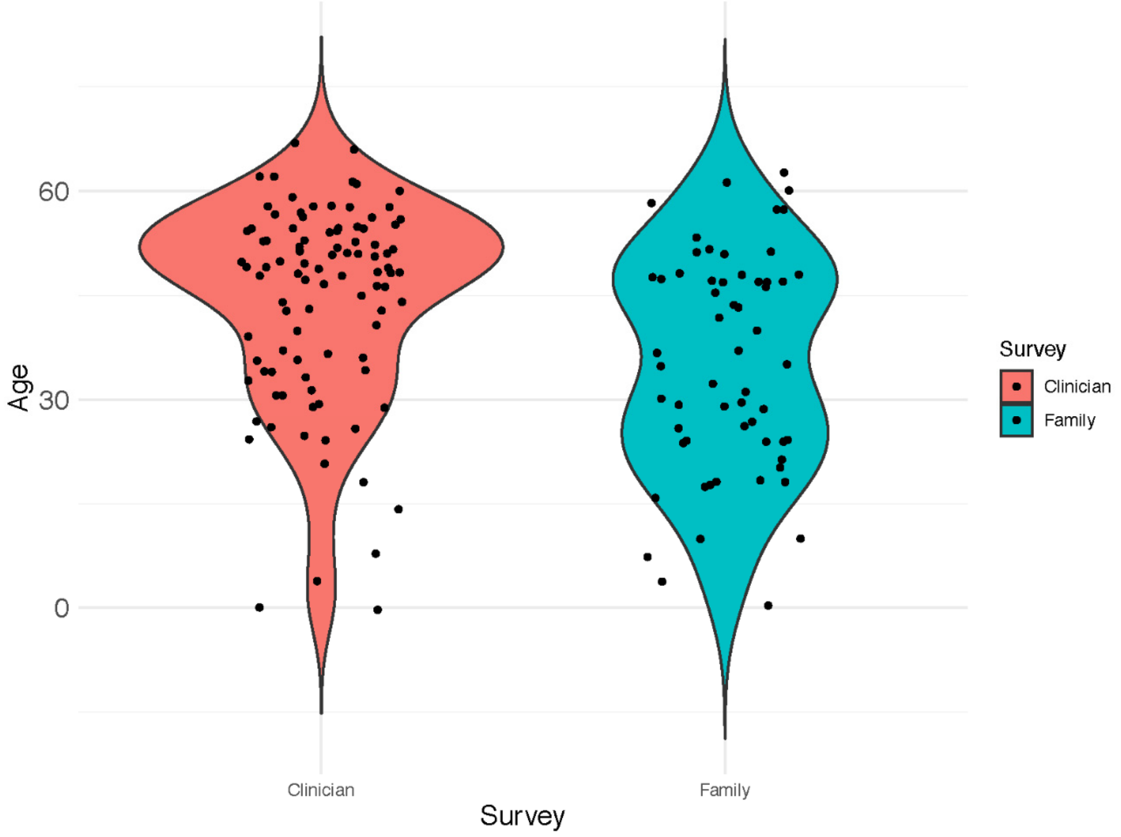

**Figure S2**. Clinical signs and symptoms of Spanish individuals with DS and COVID-19. Results are expressed as absolute frequencies (%). For details see Supplementary Table 1.

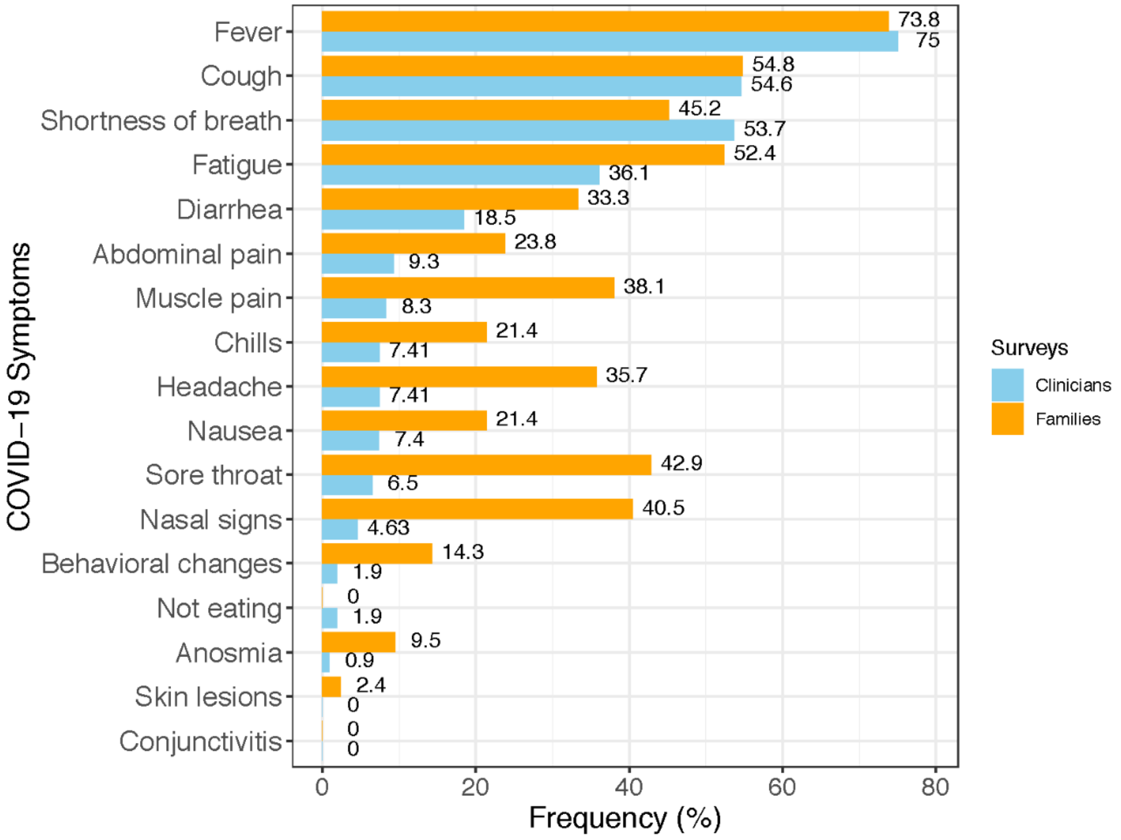

**Figure S3.** Most frequent symptoms in Spanish individuals with DS admitted to the hospital due to COVID-19. Data are represented as frequencies (percentages)

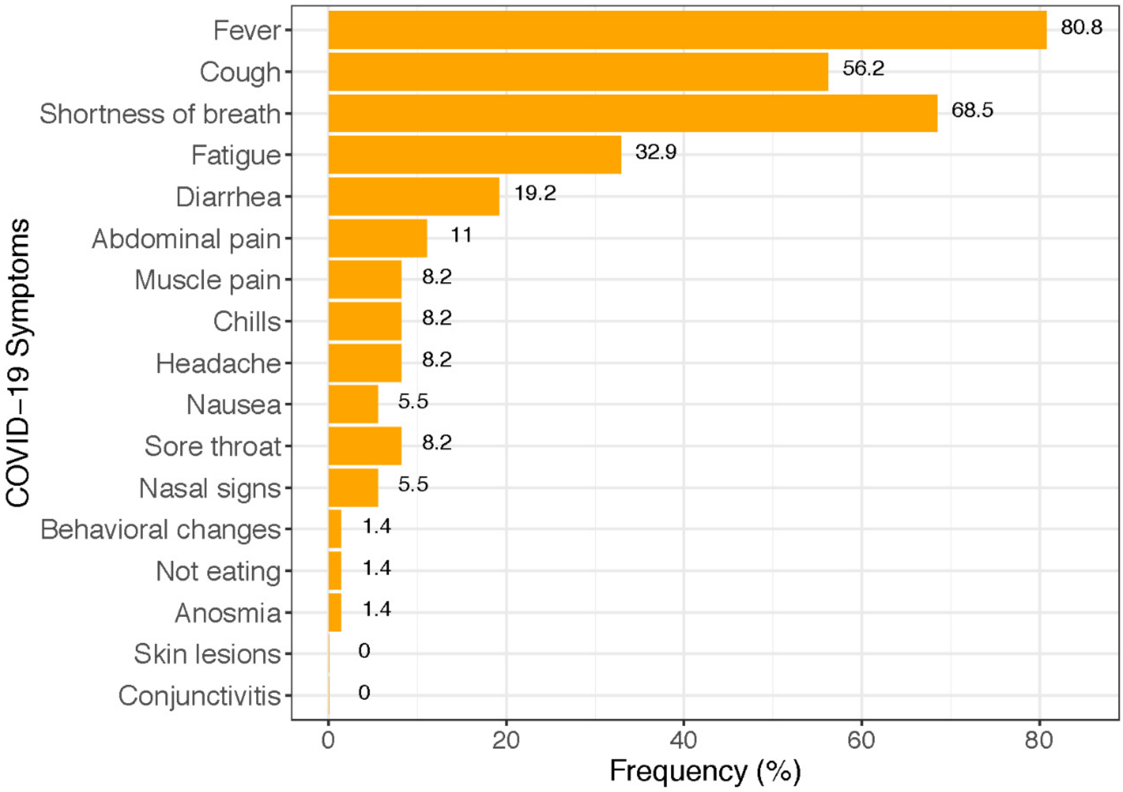

**Figure S4.** Distribution of hospitalization length of stay for individuals with DS with non-COVID-19 pneumoniae (CMBD cohort) according to age and outcome.

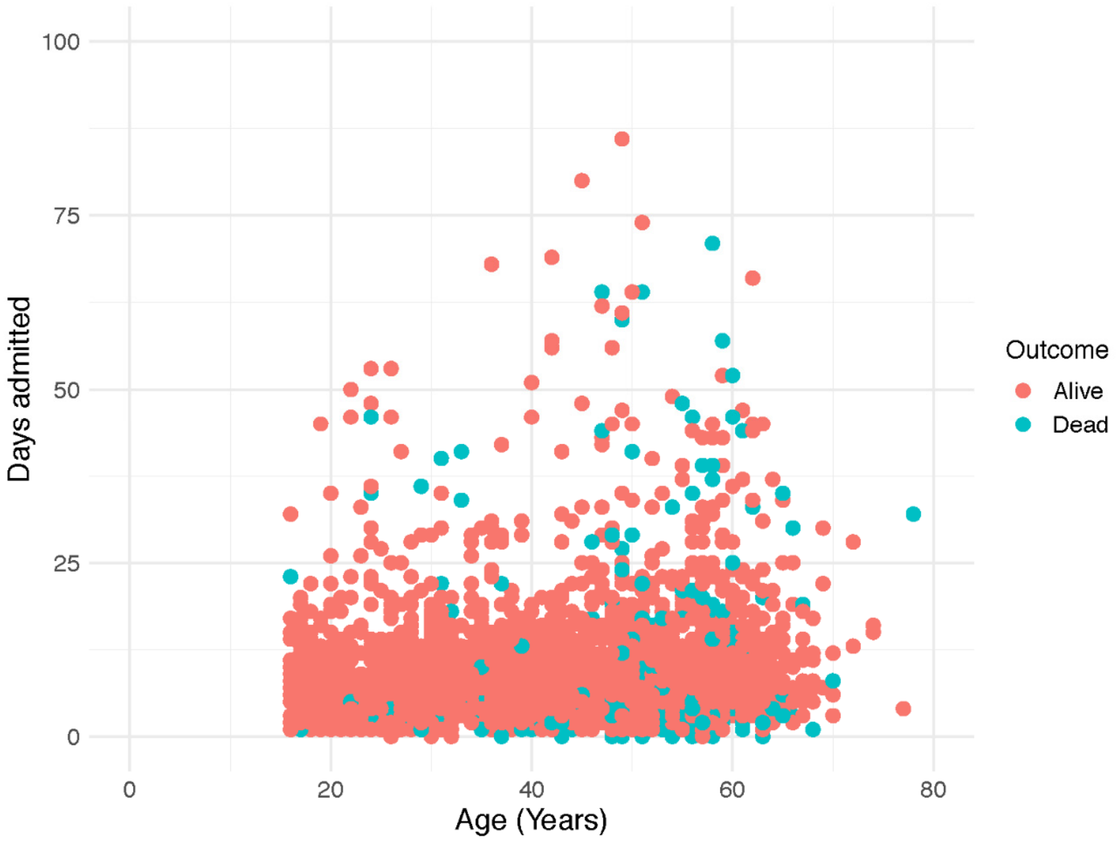

**Table S1. Socio-demographic characteristics of T21RS Spanish DS COVID-19 patients grouped by data source (reported by families or clinicians)**

| Demographics | Total n = 150 | Family surveys n = 42 | Clinician surveys n = 108 | p |
|---|---|---|---|---|
| Age (years) mean (SD) | 41·7 ± 15·6 | 33·5 ± 16.5 | 44·8 ± 14·1 | <0·001* |
| Gender (men) | 81 (54) | 20 (47·6) | 61 (56·5) | 0·46** |
| Type of trisomy | | | | |
| Full/standard | 91 (60·7) | 31 (73·8) | 60 (55·6) | |
| Mosaic | 3 (2) | 1 (2·4) | 2 (1·8) | 1** |
| Intellectual Disability | | | | |
| Borderline/Mild | 22 (14·7) | 9 (21·4) | 13 (12) | |
| Moderate/Severe | 103 (68·7) | 28 (66·7) | 75 (69·4) | 0·21** |
| Living in a residential care facility | 61/145 (42·1) | 12/42 (28·6) | 49/103 (47·6) | <0·05** |

Results are presented as mean ± SD or n (%). *Student's t test ** Chi square test

**Table S2. Relevant clinical comorbidities of T21RS Spanish DS patients with COVID-19 T21RS grouped by data source (reported by families or clinicians)**

| Comorbidities | Total n (%) | Family surveys n (%) | Clinician surveys n (%) | χ2 (p) |
|---|---|---|---|---|
| Congenital heart defect | 37/140 (26·4) | 14/41 (34·2) | 23/99 (23·2) | 0·21 |
| Psychiatric conditions | 51/141 (36·2) | 6/40 (15) | 45/101 (44·6) | < 0·001 |
| Autism | 3/150 (2) | 2/42 (4.8) | 1/108 (0·9) | 0·19 |
| Depression | 6/150 (4) | 0/42 (0) | 6/108 (5·6) | 0·19 |
| Behavioural problems | 30/150 (20) | 1/42 (2·4) | 29/108 (26·9) | < 0·001 |
| Alzheimer's disease | 34/134 (25·4) | 1/34 (2·9) | 33/100 (33) | < 0·001 |
| Thyroid disorder | 61/135 (45·2) | 13/35 (37·1) | 48/100 (48) | 0·33 |
| Seizures/epilepsy | 23/138 (16·7) | 4/34 (11·8) | 19/104 (18·3) | 0·44 |
| Blood cancer | 2/138 (1·4) | 0/33 (0) | 2/105 (1.9) | 1 |
| Obstructive sleep apnoea | 31/126 (24·6) | 7/32 (21·9) | 24/94 (25·5) | 0·81 |
| Obesity | 32/124 (25·8) | 11/36 (30·6) | 21/88 (23·9) | 0·5 |
| Hypertension | 1/134 (0·7) | 0/33 (0) | 1/101 (1) | 1 |
| Coronary heart disease | 4/134 (3) | 2/34 (5·9) | 2/100 (2) | 0·26 |
| Diabetes mellitus | 5/134 (3·7) | 0/34 (0) | 5/100 (5) | 0·33 |
| Chronic lung disease | 12/133 (9) | 1/33 (3) | 11/100 (11) | 0·29 |
| Gastrointestinal disorders | 27/136 (19·8) | 7/32 (21·9) | 20/104 (19.2) | 0.8 |

Results are presented as n (%). Denominators in each category may vary from total group sample, since missing data (not responded or "not known") in survey responses were removed from the analysis.

**Table S3.** Detailed description of clinical signs and symptoms of Spanish individuals with DS and COVID-19

| Symptoms n (%) | Clinicians (n = 108) | Families (n = 42) | $\chi2$ (p) |
|---|---|---|---|
| Fever | 81 (75) | 31 (73·9) | 1 |
| Cough | 59 (54·6) | 23 (54·8) | 1 |
| Shortness of breath | 58 (53·7) | 19 (45·2) | 0·37 |
| Fatigue | 39 (36·1) | 22 (52·4) | 0·09 |
| Diarrhoea | 20 (18·5) | 14 (33·3) | 0·08 |
| Abdominal pain | 10 (9·3) | 10 (23·8) | 0·03 |
| Muscle pain | 9 (9·3) | 16 (38·1) | < 0·001 |
| Chills | 8 (7·4) | 9 (21·4) | 0·02 |
| Headache | 8 (7·4) | 15 (37·7) | <0·001 |
| Nausea | 8 (7·4) | 9 (21·4) | 0·02 |
| Sore throat | 7 (6·5) | 18 (42·9) | <0·001 |
| Nasal signs | 5 (4·6) | 17 (40·5) | <0·001 |
| Behavioural changes | 2 (1·8) | 6 (14·3) | <0·01 |
| Not eating | 2 (1·8) | 0 (0) | 1 |
| Anosmia | 1 (0·9) | 4 (9·5) | 0.02 |
| Skin lesions | 0 (0) | 1 (2·4) | 1 |
| Conjunctivitis | 0 (0) | 0 (0) | 1 |
| Other | 15 (13·9) | 4 (9·5) | 0·59 |

Results are presented as n (%).

**Table S4**. Treatments received by admitted patients with DS and COVID-19

| Treatments | Frequency n (%) N=73 |
|---|---|
| Glucocorticoids | 26 (35·6) |
| Hydroxychloroquine | 53 (72·6) |
| Chloroquine | 2 (2·7) |
| Azithromycin | 36 (49·3) |
| Tocilizumab | 4 (5.5) |
| Melatonin | 0 (0) |
| Colchicine | 0 (0) |
| Antifungal medication | 1 (1.4) |
| Immune globulin | 0 (0) |
| Oxygen | 60 (82·2) |

The denominator in this analysis (n=73) is lower than the reported total group sample (n=86), since missing data (not responded or "not known") in survey responses were removed from the analysis. Results are presented as n (%).

**Table S5a.** Comorbidities of CMBD cohort with pneumonia.

| Comorbidity | Total<br>n | Alive<br>n (%) | Dead<br>n (%) |
|---|---|---|---|
| Alzheimer's disease | | | |
| 16-40 | 2 | 2 (100) | 0 (0) |
| >40 | 149 | 119 (79·8) | 30 (20·1) |
| Obesity | | | |
| 16-40 | 135 | 125 (92·6) | 10 (7·4) |
| >40 | 108 | 99 (91·7) | 9 (8·3) |
| Epilepsy | | | |
| 16-40 | 97 | 85 (87·6) | 12 (12.3) |
| >40 | 291 | 246 (84·5) | 45 (15·5) |

**Table S5b.** Comorbidities of Spanish DS COVID-19 patients.

| Comorbidity | Total<br>n | Alive<br>n (%) | Dead<br>n (%) |
|---|---|---|---|
| Alzheimer's disease | | | |
| 16-40 | 0 | 0 | 0 |
| >40 | 22 | 8 (36·4) | 14 (63·6) |
| Obesity | | | |
| 16-40 | 9 | 9 (100) | 0 (0) |
| >40 | 13 | 8 (61·5) | 5 (38·5) |
| Epilepsy | | | |
| 16-40 | 1 | 1 (100) | 0 (0) |
| >40 | 8 | 1 (12·5) | 7 (87·5) |

**Table S6.** In-hospital mortality of Spanish DS COVID-19 and non-COVID-19 pneumonias patients

| | **DS COVID-19 patients** | | | **Non-COVID-19 pneumonias** | | |
|---|---|---|---|---|---|---|
| Age groups | Total<br>n | Alive<br>n (%) | Dead<br>n (%) | Total<br>n | Alive<br>n (%) | Dead<br>n (%) |
| 16-40 | 23 | 23 (100) | 0 (0) | 1244 | 1185 (95·3) | 59 (4·7) |
| >40 | 62 | 39 (62·9) | 23 (30·1) | 1567 | 1353 (86·3) | 214 (13·7) |

Results are presented as n or n (%).
